# Supplementary material for: Differences in self-reported health between cardiac arrest survivors with good cerebral performance and survivors with moderate cerebral disability: a nationwide register study
Source: BMJ Open. 2022 Jul 11;12(7):e058945. doi: 10.1136/bmjopen-2021-058945 (PMC9274516; doi:10.1136/bmjopen-2021-058945)
Supplement: Author's manuscript [file bmjopen-2021-058945.draft_revisions.pdf]

BMJ Open is committed to open peer review. As part of this commitment we make the peer review history of every article we publish publicly available.

When an article is published we post the peer reviewers' comments and the authors' responses online. We also post the versions of the paper that were used during peer review. These are the versions that the peer review comments apply to.

The versions of the paper that follow are the versions that were submitted during the peer review process. They are not the versions of record or the final published versions. They should not be cited or distributed as the published version of this manuscript.

BMJ Open is an open access journal and the full, final, typeset and author-corrected version of record of the manuscript is available on our site with no access controls, subscription charges or pay-per-view fees (http://bmjopen.bmj.com).

If you have any questions on BMJ Open's open peer review process please email info.bmjopen@bmj.com

Self-reported health in cardiac arrest survivors with favourable neurological outcome – a nationwide comparative register study

Journal:	*BMJ Open*
Manuscript ID	bmjopen-2021-058945
Article Type:	Original research
Date Submitted by the Author:	02-Nov-2021
Complete List of Authors:	Larsson, Karin; Linkoping University Department of Health Medicine and Caring Sciences,<br>Hjelm, Carina; Linkoping University Department of Health Medicine and Caring Sciences<br>Lilja, Gisela ; Lund University, Department of Clinical Sciences<br>Stromberg, Anna; Linkoping University Department of Health Medicine and Caring Sciences; Department of cardiology, Linkoping University<br>Årestedt, Kristofer; Linnaeus University Faculty of Health and Life Sciences; The Research Section, Region Kalmar County
Keywords:	Adult cardiology < CARDIOLOGY, Coronary heart disease < CARDIOLOGY, Rehabilitation medicine < INTERNAL MEDICINE, INTENSIVE & CRITICAL CARE

SCHOLARONE™
Manuscripts

I, the Submitting Author has the right to grant and does grant on behalf of all authors of the Work (as defined in the below author licence), an exclusive licence and/or a non-exclusive licence for contributions from authors who are: i) UK Crown employees; ii) where BMJ has agreed a CC-BY licence shall apply, and/or iii) in accordance with the terms applicable for US Federal Government officers or employees acting as part of their official duties; on a worldwide, perpetual, irrevocable, royalty-free basis to BMJ Publishing Group Ltd ("BMJ") its licensees and where the relevant Journal is co-owned by BMJ to the co-owners of the Journal, to publish the Work in this journal and any other BMJ products and to exploit all rights, as set out in our licence.

The Submitting Author accepts and understands that any supply made under these terms is made by BMJ to the Submitting Author unless you are acting as an employee on behalf of your employer or a postgraduate student of an affiliated institution which is paying any applicable article publishing charge ("APC") for Open Access articles. Where the Submitting Author wishes to make the Work available on an Open Access basis (and intends to pay the relevant APC), the terms of reuse of such Open Access shall be governed by a Creative Commons licence – details of these licences and which Creative Commons licence will apply to this Work are set out in our licence referred to above.

Other than as permitted in any relevant BMJ Author's Self Archiving Policies, I confirm this Work has not been accepted for publication elsewhere, is not being considered for publication elsewhere and does not duplicate material already published. I confirm all authors consent to publication of this Work and authorise the granting of this licence.

For peer review only

Self-reported health in cardiac arrest survivors with favourable neurological outcome – a nationwide comparative register study

Karin Larsson RN PhD student[1], Carina Hjelm RN PhD[1], Gisela Lilja OT PhD[2], Anna Strömberg RN Professor[1,3], Kristofer Årestedt RN Professor[4,5]

1 Department of Health, Medicine and Caring Sciences, Linköping University, SE-581 83, Linköping, Sweden
2 Lund University, Skane University Hospital, Department of Clinical Sciences Lund, Neurology, SE-221 85, Lund, Sweden
3 Department of Cardiology, Linköping University, SE-581 83, Linköping, Sweden
4 Faculty of Health and Life Sciences, Linnaeus University, SE-391 82, Kalmar, Sweden
5 The Research Section, Region Kalmar County, SE-391 26, Kalmar, Sweden

Corresponding author: Karin Larsson, RN, PhD student
Department of Health, Medicine and Caring Sciences, Linköping University
SE- 581 83, Linköping
Sweden
Email: karin.larsson@liu.se
Phone: +46 13 284606

Word count Paper: 2933, Word count Abstract: 218
Figures: 1
Tables: 5

For peer review only

For peer review only - http://bmjopen.bmj.com/site/about/guidelines.xhtml

ABSTRACT

Objective: The aim was to compare self-reported health of cardiac arrest survivors with good cerebral performance (CPC 1) and survivors with moderate cerebral disability (CPC 2).

Methods: This comparative register study was based on nationwide data from the Swedish Register of Cardiopulmonary Resuscitation. The study included 2,058 in-hospital and out-of-hospital cardiac arrest survivors with good cerebral performance or survivors with moderate cerebral disability, 3-6 months post cardiac arrest. Survivors completed a questionnaire including the Hospital Anxiety and Depression Scale (HADS) and EQ-5D five-levels (EQ-5D-5L). Data were analysed using ordinal and linear regression models.

Results: For all survivors, the prevalence of anxiety and depression symptoms measured by the HADS was 14% and 13% respectively. Using the EQ-5D-5L, the cardiac arrest survivors reported most health problems relating to pain/discomfort (57%), followed by anxiety/depression (47%), usual activities (46%), mobility (40%), and self-care (18%). Compared to the survivors with good cerebral performance, survivors with moderate cerebral disability reported significantly higher symptom levels of anxiety and depression measured with HADS, and poorer health in all dimensions of the EQ-5D-5L after adjusting for age, sex, place of cardiac arrest, aetiology, and initial rhythm ($p<0.001$).

Conclusions: These findings stress the importance of screening for health problems in all cardiac arrest survivors to identify those in need of professional support and rehabilitation, independent on neurological outcome.

Keywords: Anxiety, Cerebral performance, Depression, Health status, Heart arrest, Self-reported health

For peer review only - http://bmjopen.bmj.com/site/about/guidelines.xhtml

Strengths and limitations of this study:

- The study includes a large sample of cardiac arrest survivors (n=2,058), both hospital and out-of-hospital cardiac arrest survivors with a favourable neurological outcome.
- The data is taken from the Swedish Register of Cardiopulmonary Resuscitation, a nationwide registry with nearly complete coverage of all cardiac arrest in Sweden where cardiopulmonary resuscitation is started.
- The cross-sectional design omits any causal conclusions.
- The reason for dropouts is un-known and non-response bias can therefore not be excluded.

For peer review only

For peer review only - http://bmjopen.bmj.com/site/about/guidelines.xhtml

INTRODUCTION

Cardiac arrest (CA) is a global health problem with poor survival. The survival rate varies across studies and countries but is, according to recent studies, around 20% for in-hospital cardiac arrest (IHCA)[1] and 10% for out-of-hospital cardiac arrest (OHCA).[2] CA results rapidly in hypoxic-ischaemic brain damage[3] and the neurological outcome is therefore an important outcome, commonly assessed with the Cerebral Performance Category (CPC) scale.[4] The CPC classification is made by health care professionals on a scale ranging from 1 to 5, where CPC 1 and 2 (i.e., good cerebral performance and moderate cerebral disability) are generally defined as a favourable neurological outcome.[5-8] In contrast CPC 3 to 5 (severe cerebral disability to brain death) are defined as unfavourable neurological outcomes and imply dependency on others.[4,9]

Health-related quality of life (HRQoL) reflects the outcome after a CA from the survivor's perspective and has been recommended as one of the core outcomes post CA.[10] Health is a broad concept defined by WHO as "a state of complete physical, mental and social well-being and not merely the absence of disease or infirmity".[11] Previous studies have shown that CA survivors in general report good health.[12-14] In fact, one comparative study showed that survivors reported even higher levels of health compared to a sex- and age-matched normal population.[14] However, a significant minority of the CA survivors report severe health problems, particularly with symptoms of anxiety and depression.[12-13,15]

Although cerebral performance and self-reported health are both important outcomes, the association between them is not well explored among CA survivors. A study by Geri et al.[16] found that survivors with good cerebral performance (CPC 1) reported better health compared to survivors with moderate or severe cerebral disability (CPC 2 and 3). No study has been

For peer review only - http://bmjopen.bmj.com/site/about/guidelines.xhtml

identified that has specifically investigated differences in self-reported health of CA survivors with good cerebral performance (CPC 1) and moderate cerebral disability (CPC 2). As these survivors are defined as having a favourable neurological outcome, there is a risk that health problems could be overlooked. Therefore, the aim of the present study was to compare self-reported health between cardiac arrest survivors with good cerebral performance (CPC 1) and survivors with moderate cerebral disability (CPC 2).

METHODS

Design

This comparative registry study was based on data from the Swedish Register of Cardiopulmonary Resuscitation. Overall, the data is the same as was used in a study by Djärv et al.[15] that focused on the differences in HRQoL between IHCA and OHCA survivors. However, the present study does not include survivors with CPC >2.

Setting

The Swedish Register of Cardiopulmonary Resuscitation (https://shlrsjh.registercentrum.se/) is a nationwide quality register consisting of two parts: IHCA and OHCA. All cardiac arrests are registered when cardiopulmonary resuscitation (CPR) has been started. The register covers almost 100% of OHCA and a majority of IHCA in Sweden. All survivors 18 years or older who are still alive 3–6 months post CA are screened for eligibility to complete a questionnaire about self-reported health by resuscitation coordinators or cardiac rehabilitation nurses. Unwillingness to participate, severe cognitive dysfunction, language difficulties, and severe physical and/or psychological difficulties (according to the latest medical file) were criteria for exclusion in the register during the study period. A letter is sent to the CA survivors, including information about the registry, two questionnaires (the Hospital Anxiety

For peer review only

and Depression Scale and EQ-5D-5L), and an invitation for a structured interview by phone to collect information about their outcome. The CA survivors are instructed to complete the questionnaire during the interview. The interview is performed by the resuscitation coordinators or cardiac rehabilitation nurses. At the time of the interview, cerebral performance is also assessed based on the interview, using the CPC score.

Sample and procedure

The present study included registry data collected between 1st of January 2014 and 31st of December 2017. During this period, 2,141 CA survivors, 772 OHCA (response rate 54.6%) and 1,369 IHCA (response rate 57.4%), had completed the questionnaire. Of these, 57 survivors had CPC ≥3 and 26 (1.2%) had missing data on the CPC assessment and were therefore excluded. Thus, the final sample consisted of 2,058 survivors.

Outcome measures of self-reported health

Symptoms of anxiety and depression

The Hospital Anxiety and Depression Scale (HADS) is developed to measure symptoms of anxiety and depression during the last week. The instrument consists of two sub-scales, HADS anxiety and HADS depression. Each subscale includes seven items, which all have four response options (0-3). The responses to the items in each subscale are summed to a subscale score that can vary between 0 and 21; higher scores indicate higher symptom level.[17] The following cut-off values have been recommended: 0-7 normal range, 8-10 suggested presence of anxiety and/or depression and 11-21 probable presence of anxiety and/or depression.[18] Both subscale scores and cut-off scores were used in the present study. The instrument has previously been used in CA research[12,19] and has shown good

For peer review only - http://bmjopen.bmj.com/site/about/guidelines.xhtml

measurement properties.[20] Cronbach's alpha was 0.88 and 0.85 for the anxiety and depression subscales respectively in the present study.

Health status

The EQ-5D-5L is a generic instrument to measure health status and includes three parts: EQ-5D-5L descriptive system, EQ VAS and EQ-5D value index (the latter not used in the present study).[21] The EQ-5D-5L descriptive system consists of five health dimensions: mobility, self-care, usual activities, pain/discomfort, and anxiety/depression. Each dimension has five response options, referred to as levels, ranging from no problems (1) unable to do/extreme problems (5). These scores reflect the intensity of the health problems. The five levels can also be dichotomised into no problem (1) and problems (2-5), reflecting the prevalence of the health problems. Both prevalence and intensity scoring are used in the present study. The EQ VAS is a measure of overall health and graded on a 0-100 scale, where 0 represents worst possible health and 100 best possible health.[21] The EQ-5D-5L has been recommended as a core outcome in CA research[10] and has in previous studies shown good measurement properties in different patient groups, e.g. stroke and chronic obstructive pulmonary disease.[22-23]

Statistical analysis

To present the characteristics of the survivors and the variables, descriptive statistics were used. To compare self-reported health between CA survivors with good cerebral performance (CPC 1) and survivors with moderate cerebral disability (CPC 2), chi-square test was used for nominal data and Mann-Whitney U test was used for ordinal data.

For peer review only - http://bmjopen.bmj.com/site/about/guidelines.xhtml

Multiple linear regression analyses were used to investigate associations between cerebral function (CPC as the explanatory variable) and self-reported health (the EQ VAS and HADS as outcome variables). Multiple ordinal logistic regression analyses were used to handle the ordinal nature of data in the EQ-5D-5L health dimensions. Cerebral function (CPC 1=0, CPC 2=1) was included as an explanatory variable and the EQ-5D-5L health dimensions as outcome variables. Both the linear and ordered logistic regression models were adjusted for covariates that are commonly used in cardiac arrest research;[12,15] age, sex (male=0, female=1), place of CA (IHCA=0, OHCA=1), aetiology (heart disease=1, other=0), and initial rhythm. Initial rhythm was coded as shockable rhythm (VF/VT) and non-shockable rhythm (PEA and asystole). To handle the large number of missing data in initial rhythm (n=341), they were coded within a third 'unknown' group. Thus, initial rhythm was included as a dummy coded variable in the regression models, with shockable rhythm as a reference category.

The level of statistical significance was set at $p<0.05$ and the data were analysed using SPSS statistics 24 (IBM Corp., Armonk, NY, USA) and Stata 16.1 (StataCorp LLC, College Station, TX, USA).

RESULTS

Survivor characteristics

The final sample consisted of 2,058 survivors: 1,309 IHCA survivors and 749 OHCA survivors. The median age was 68 years (range=59-75), and the majority were male (n=1,438, 70%). Most survivors were reported to have good cerebral performance (CPC 1, n=1,838, 89%). A majority of the cardiac arrests were witnessed (n=1,902, 94%) and caused by heart disease (n=1,472, 73%). About three out of four survivors had an initial shockable rhythm

For peer review only - http://bmjopen.bmj.com/site/about/guidelines.xhtml

(n=1,242, 72%), and 63% (n=1,297) were defibrillated. The median time to defibrillation was 2 minutes (q1-q3=1-8). Compared to survivors with moderate cerebral disability, those with good cerebral performance were significantly younger (p=0.002), more often male (p=0.009), had more often an initial shockable rhythm (p<0.001), were more often defibrillated (p=0.002) and more often had heart disease as the cause of CA (p<0.001). In addition, the share of OHCA (p=0.011) was larger in the group of survivors with good cerebral performance than in the group of survivors with moderate cerebral disability. The difference in time to defibrillation and whether the CA was witnessed did not show any significant difference between survivors with good cerebral performance and survivors with moderate cerebral disability (Table 1).

Symptoms of anxiety and depression (HADS)

Subscale-scores for the HADS showed that the survivors generally reported low symptom levels of anxiety (Mdn=2, q1-q3=0-5) and depression (Mdn=2, q1-q3=1-5). However, survivors with moderate cerebral disability reported significantly higher symptom levels of both anxiety (ΔMdn=2, p<0.001) and depression (ΔMdn=3, p<0.001) than survivors with good cerebral performance (Table 2). Based on the multiple linear regression analyses, these differences remained when age, sex, place of CA, aetiology, and initial rhythm were adjusted for (p<0.001). The regression model explained 8% of the total variance in symptoms of anxiety and 7% in symptoms of depression (Table 3).

Using the cut-off scores for the HADS, 280 (14%) of all the CA survivors reported having suggested or probable presence of anxiety and 261 (13%) suggested or probable presence of depression. However, the share of survivors with suggested or probable presence of anxiety and depression was significantly higher among survivors with moderate cerebral disability

For peer review only - http://bmjopen.bmj.com/site/about/guidelines.xhtml

than in survivors with good cerebral performance ($p<0.001$). Among the survivors with moderate cerebral disability, 53 (24%) reported having symptoms of anxiety and 64 (29%) symptoms of depression (Table 4).

Health status (EQ-5D-5L and EQ VAS)

Using the EQ-5D-5L descriptive systems, the CA survivors reported pain/discomfort as the most prevalent health problem (n=1172, 56.9%), followed by anxiety/depression (n=971, 47.2%), usual activities (n=952, 46.3%), mobility (n=822, 40.0%) and self-care (n=365, 17.7%). All health problems were significantly more prevalent among survivors with moderate cerebral disability than survivors with good cerebral performance ($p<0.001$) (Fig.1).

Although health problems were common, the CA survivors reported, on average, low intensity levels of all health problems (Mdn=1-2). However, the survivors with moderate cerebral disability reported significantly higher intensity levels for all health dimensions compared to the survivors with good cerebral performance ($p<0.001$). Survivors with good cerebral performance reported the highest levels of pain/discomfort (Mdn=2, q1-q3=1-2) while survivors with moderate cerebral disability reported the highest levels of usual activities (Mdn=3, q1-q3=2-4) (Table 2). Based on the ordinal regression analyses, these group differences remained when age, sex, place of CA, aetiology, and initial rhythm were included as adjusting covariates ($p<0.001$). The pseudo R^2 values (McFadden) ranged between 0.02 and 0.08 (Table 5).

The CA survivors reported overall a median score of 75 (q1-q3= 60-85) on the EQ VAS; survivors with moderate cerebral disability reported significantly worse health status than survivors with good cerebral performance (ΔMdn=15, $p<0.001$) (Table 2). Based on the

For peer review only - http://bmjopen.bmj.com/site/about/guidelines.xhtml

multiple linear regression analysis, this group difference remained in the adjusted model ($p<0.001$). The regression model explained 10% of the total variance in EQ VAS (Table 3).

DISCUSSION

To the best of our knowledge, this is the largest study to have compared self-reported health between CA survivors with good cerebral performance (CPC 1) and survivors with moderate cerebral disability (CPC 2). Although these groups are commonly defined as having a favourable neurological outcome after their CA, the results show that survivors with moderate cerebral disability reported significantly more health problems, measured by the HADS and EQ-5D-5L, compared to survivors with good cerebral performance. These differences remained after adjustment for age, sex, place of CA, aetiology, and initial rhythm.

The survivors generally reported low symptom levels of anxiety and depression, but a great difference was shown between those with moderate cerebral disability and those with good cerebral performance. The prevalence of anxiety and depression in the present study corresponds well with a systematic review about psychological health post CA[24]. Most of the included studies that had used the HADS reported a prevalence of 13-14% of both anxiety and depression, thus levels were similar to those shown for the whole sample in the present study. It should be noted that the prevalence of anxiety and depression in the present study was significantly higher in survivors with moderate cerebral disability. The prevalence of anxiety in this group corresponds with a Swedish study of 278 OHCA survivors treated with target temperature management, at six-months' follow-up.[25] However, the prevalence of depression in that study was lower compared to the survivors with moderate cerebral disability in the present study. This finding was unexpected as all patients in the temperature

For peer review only - http://bmjopen.bmj.com/site/about/guidelines.xhtml

study, in contrast to the present study, had received intensive care and thereby experienced a more complicated disease trajectory.

The prevalence of depression was more common than anxiety in survivors with moderate cerebral disability and the opposite situation was shown for survivors with good cerebral performance. Included studies in a systematic review by Wilder Schaaf et al. reported higher prevalence of anxiety than depression, or equal levels, in CA survivors. However, the opposite situation was not reported. It should be noted that only OHCA survivors were included in that review, the number of included studies was small (n=11), and the sample size in all included studies was small (21-168 survivors). In addition, different measures were used to assess symptoms of anxiety and depression, and only four studies used the HADS.[24] The HADS' ability to differentiate between anxiety and depression has been questioned, and a review about the psychometric properties concluded that the subscales therefore should be compared with caution.[26]

Unfortunately, there are no Swedish normative data for symptoms of anxiety and depression measured by the HADS. Compared to normative data for anxiety and depression measured by the HADS in the UK,[27] the prevalence of anxiety was less common while depression was more common in survivors with good cerebral performance. In addition, the prevalence of having suggested or probable presence of anxiety and depression was more common in survivors with moderate cerebral disability compared to normative data from the UK. It should be noted that normative data from the UK may differ from the general population in Sweden. However, the results indicate that symptoms of anxiety and depression need to be acknowledged also in CA survivors with favourable neurological outcome.

For peer review only - http://bmjopen.bmj.com/site/about/guidelines.xhtml

Survivors with moderate cerebral disability also reported more problems with their physical health, measured with the EQ-5D-5L, compared to survivors with good cerebral performance. Age and comorbidity may explain these differences as survivors with moderate cerebral disability were significantly older and more often suffered ICHA compared to survivors with good cerebral performance. However, the multiple regression analyses in the present study show that age could not explain these differences. Unfortunately, data of comorbidity is not registered for OHCA survivors in the Swedish Register of Cardiopulmonary Resuscitation and could therefore not be examined in the present study. It is likely that comorbidity is an important confounder as previous studies have shown that comorbidity is related to self-reported health in CA survivors[28] and is likely to be related to cerebral performance as well. Comorbidity should therefore be considered in future research.

Methodological limitations

The study has some limitations that need to be considered. No causal relationship can be drawn due to the cross-sectional design. The no-response rate was acceptable; however, the reasons for not participating are not known. It is a potential risk that the response rate is lower among survivors with moderate cerebral disability and those with poor self-reported health. In addition, survivors with severe physical and/or psychological difficulties were excluded from the third registration. Therefore, we cannot be sure that the participants are representative of the entire population of CA survivors. Information about survivors' cerebral performance before CA is unknown and therefore, we do not know if the impaired cerebral performance among those survivors who were classified according to CPC 2 was caused by the CA. The use of CPC may be criticized as it did not take to account prior disability. Of this reason, other measures like the Modified Ranking Scale and extended Glasgow Outcome Scale are commonly recommended to be used in cardiac arrest survivors. However, the correlation

For peer review only - http://bmjopen.bmj.com/site/about/guidelines.xhtml

between these outcome measures is high.[10,29] To exclude the risk that the assessment of the CPC at the follow-up assessment would be affected by the survivors' self-reported health during the interview, the agreement with CPC at hospital discharge was examined. The absolute agreement was 81% and the risk for that is therefore rather small. The HADS and EQ-5D-5L were used to measure self-reported health as they are recommended in international guidelines for post CA care.[10,30] However, neither HADS nor EQ-5D-5L are disease specific for CA survivors and might therefore not capture specific health problems related to surviving a life-threatening condition such as CA. Even so, the instruments capture important health problems, and early screening could be a way to identify survivors at risk of low self-reported health, also in survivors with favourable neurological outcome, particularly in survivors with moderate cerebral disability.

CONCLUSIONS

This study contributes with important knowledge about cerebral performance and self-reported health in CA survivors with favourable neurological outcome. Survivors with moderate cerebral disability (CPC 2) reported significantly lower levels of self-reported health compared to survivors with good cerebral performance (CPC 1). These findings stress the importance of screening for health problems in all cardiac arrest survivors to identify those in need of professional support and rehabilitation, independent of neurological outcome. Until there are CA-specific instruments to measure self-reported health, the HADS and EQ-5D-5L serve as adequate screening instruments.

Acknowledgements

We would like to thank all the participating survivors in the Swedish Register of Cardiopulmonary Resuscitation.

For peer review only - http://bmjopen.bmj.com/site/about/guidelines.xhtml

Contributors

KL: contributed to the conception of the design, the acquisition of the data, analysis, and interpretation of data, drafting and revising the work critically for important intellectual content and approval of the final manuscript.

CH: contribute to the conception of the design, interpretation of data, drafting and revising the work critically for important intellectual content and approval of the final manuscript.

GL: contributed to the conception of the design, revising the work critically for important intellectual content and approval of the final manuscript.

AS: contributed to the conception of the design, revising the work critically for important intellectual content and approval of the final manuscript.

KÅ: contributed to the conception of the design, the acquisition of the data, analysis, and interpretation of data, drafting and revising the work critically for important intellectual content and approval of the final manuscript.

For peer review only

Funding

This work was supported by The Medical Research Council of Southeast Sweden (FORSS-745341 and FORSS-939845) and Region Östergötland, Linköping University (RÖ-920281), Sweden.

Competing interests

None to declare.

Patient consent for publication

Not required.

Ethics approval

The study was approved by the Regional Ethical Review Board in Linköping, Sweden (No. 2018/480-21).

For peer review only

For peer review only - http://bmjopen.bmj.com/site/about/guidelines.xhtml

References

1 Andersen LW, Holmberg MJ, Berg KM, et al. In-hospital cardiac arrest: a review. *JAMA Intern Med* 2019;321:1200-10 doi:10.1001/jama.2019.1696 [published Online First: 25 April 2019].

2 Shijiao Y, Yong G, Nan J, et al. The global survival rate among adult out-of-hospital cardiac arrest patients who received cardiopulmonary resuscitation: a systematic review and meta-analysis. *Crit Care* 2020;24:1-13 doi:10.1186/s13054-020-2773-2.

3 Busl KM, Greer DM. Hypoxic-ischemic brain injury: pathophysiology, neuropathology and mechanisms. *NeuroRehabilitation* 2010;26:5-13 doi:10.3233/NRE-2010-0531.

4 Cummins RO, Chamberlain DA, Abramson NS, et al. Recommended guidelines for uniform reporting of data from out-of-hospital cardiac arrest: The Utstein style: a statement for health professionals from a task force of the American Heart Association, the European Resuscitation Council, and Heart and Stroke Foundation of Canada, and the Australian Resuscitation Council. *Circulation* 1991;84:960-75 doi:10.1161/01.CIR.84.2.960.

5 Akin M, Garcheva V, Sieweke J-T, et al. Neuromarkers and neurological outcome in out-of-hospital cardiac arrest patients treated with therapeutic hypothermia–experience from the HAnnover COoling REgistry (HACORE). *PloS one* 2021;16:e0245210 doi:10.1371/journal.pone.0245210.

6 Reynolds EC, Zenasni Z, Harrison DA, et al. How do information sources influence the reported Cerebral Performance Category (CPC) for in-hospital cardiac arrest survivors? An observational study from the UK National Cardiac Arrest Audit (NCAA). *Resuscitation* 2019;114:19-23 doi:10.1016/j.resuscitation.2019.05.035 [published Online First: 12 June 2019].

For peer review only - http://bmjopen.bmj.com/site/about/guidelines.xhtml

7 Eertmans W, Tran TMP, Genbrugge C, et al. A prediction model for good neurological outcome in successfully resuscitated out-of-hospital cardiac arrest patients. *Scand J Trauma Resusc Emerg Med* 2018;26:93 doi:10.1186/s13049-018-0558-2.

8 Gräsner J-T, Herlitz J, Tjelmeland IBM, et al. European Resuscitation Council Guidelines 2021: epidemiology of cardiac arrest in Europe. *Resuscitation* 2021;161:61-79 doi:10.1016/j.resuscitation.2021.02.007 [published Online First: 24 March 2021].

9 Jennett B, Bond M. Assessment of outcome after severe brain damage. A practical scale. *Lancet* 1975;305:480-4 doi:10.1016/S0140-6736(75)92830-5.

10 Haywood K, Whitehead L, Nadkarni VM, et al. COSCA (Core Outcome Set for Cardiac Arrest) in adults: an advisory statement from the International Liaison Committee on Resuscitation. *Cirkulation* 2018;137:e783-e801. doi:10.1161/CIR.0000000000000562 [published Online First: 26 April 2018].

11 WHO. Preamble to the Constitution of WHO as adopted by the International Health Conference, New York, 19 June - 22 July 1946; signed on 22 July 1946 by the representatives of 61 States (Official Records of WHO, no. 2, p. 100) and entered into force on 7 April 1948.

12 Israelsson J, Bremer A, Herlitz J, et al. Health status and psychological distress among in-hospital cardiac arrest survivors in relation to gender. *Resuscitation* 2017;114:27-33 doi:10.1016/j.resuscitation.2017.02.006 [published Online First: 17 February 2017].

13 Moulaert VRM, van Heugten CM, Gorgels TPM, et al. Long-term outcome after survival of a cardiac arrest: a prospective longitudinal cohort study. *Neurorehabil Neural Repair* 2017;31:530-9 doi:10.1177/1545968317697032.

For peer review only - http://bmjopen.bmj.com/site/about/guidelines.xhtml

14 Tiainen M, Vaahersalo J, Skrifvars MB, et al. Surviving out-of-hospital cardiac arrest: the neurological and functional outcome and health-related quality of life one year later. *Resuscitation* 2018;129:19-23 doi:10.1016/j.resuscitation.2018.05.011 [published Online First: 15 May 2018].

15 Djärv T, Bremer A, Herlitz J, et al. Health-related quality of life after surviving an out-of-hospital compared to an in-hospital cardiac arrest: A Swedish population-based registry study. *Resuscitation* 2020;151:77-84 doi:10.1016/j.resuscitation.2020.04.002 [published Online First: 12 April 2020].

16 Geri G, Bougouin W, Champigneulle B, et al. Predictors of long-term functional outcome and health-related quality of life after out-of-hospital cardiac arrest. *Resuscitation* 2017;113:77-82 doi:10.1016/j.resuscitation.2017.01.028 [published Online First: 12 February 2017].

17 Zigmond AS, Snaith RP. The hospital anxiety and depression scale. *Acta Psychiatr Scand* 1983;67:361-70 doi:10.1111/j.1600-0447.1983.tb09716.x.

18 Snaith RP. The Hospital Anxiety And Depression Scale. *Health Qual Life Outcomes* 2003;1:29-4 doi:10.1186/1477-7525-1-29.

19 Buanes EA, Flaatten H, Husby T, et al. Cognitive function and health-related quality of life four years after cardiac arrest. *Resuscitation* 2015;89:13-8 doi:10.1016/j.resuscitation.2014.12.021 [published Online First: 14 January 2015].

20 Djukanovic I, Carlsson J, Årestedt K. Is the Hospital Anxiety and Depression Scale (HADS) a valid measure in a general population 65-80 years old? A psychometric evaluation study. *Health Qual Life Outcomes* 2017;15:1-10 doi:10.1186/s12955-017-0759-9.

For peer review only

For peer review only - http://bmjopen.bmj.com/site/about/guidelines.xhtml

21 EuroQol. EuroQol - a new facility for the measurement of health-related quality of life. Health policy (Amsterdam, Netherlands) 1990;16:199-208 doi:10.1016/0168-8510(90)90421-9.

22 Nolan CM, Longworth L, Lord J, et al. The EQ-5D-5L health status questionnaire in COPD: validity, responsiveness and minimum important difference. *Thorax* 2016;71: 493-500 doi:10.1136/thoraxjnl-2015-207782 [published Online First: 30 March 2016].

23 Lu W-S, Huang S-L, Yang J-F, et al. Convergent validity and responsiveness of the EQ-5D utility weights for stroke survivors. *J Rehabil Med* 2016;48:346-51 doi: 10.2340/16501977-2069.

24 Wilder Schaaf KP, Artman LK, Peberdy MA, et al. Anxiety, depression, and PTSD following cardiac arrest: a systematic review of the literature. *Resuscitation* 2013;84:873-7 doi:10.1016/j.resuscitation.2012.11.021 [published Online First: 29 November 2012].

25 Lilja G, Nilsson G, Nielsen N, et al. Anxiety and depression among out-of-hospital cardiac arrest survivors. *Resuscitation* 2015;97:68-75 doi:10.1016/j.resuscitation.2015.09.389 [published Online First: 9 October 2015].

26 Cosco TD, Doyle F, Ward M, McGee H. Latent structure of the Hospital Anxiety And Depression Scale: a 10-year systematic review. *J Psychosom Res* 2012;72:180-4 doi:10.1016/j.jpsychores.2011.06.008 [published Online First: 14 September 2011].

27 Breeman S, Cotton S, Fielding S, et al. Normative data for the Hospital Anxiety and Depression Scale. *Qual Life Res* 2015;24:391-8 doi:10.1007/s11136-014-0763-z [published Online First: 27 July 2014].

28 Andrew E, Nehme Z, Bernard S, et al. The influence of comorbidity on survival and long-term outcomes after out-of-hospital cardiac arrest. *Resuscitation* 2017;110:42-7 doi:10.1016/j.resuscitation.2016.10.018 [published Online First: 2 November 2016].

For peer review only - http://bmjopen.bmj.com/site/about/guidelines.xhtml

29 Raina KD, Rittenberger JC, Holm MB, et al. Functional outcomes: one year after a cardiac arrest. *Biomed Res Int* 2015 doi:10.1155/2015/283608 [published Online First 3 September 2015].

30 Nolan JP, Sandroni C, Böttiger BW, et al. European Resuscitation Council and European Society of Intensive Care Medicine Guidelines 2021: post-resuscitation care. *Resuscitation* 2021;161:220-69 doi:10.1007/s00134-021-06368-4 [published Online First: 25 March 2021].

For peer review only

For peer review only - http://bmjopen.bmj.com/site/about/guidelines.xhtml

Table 1. Background data of the survivors with good cerebral performance (CPC 1) and survivors with moderate cerebral disability (CPC 2).

	Valid n	All n=2,058	CPC 1 n=1,838	CPC 2 n=220	p-value
Age (years), Mdn (q1-q3)	2,057	68 (59-75)	68 (58-75)	71 (62-79)	0.002 [a]
Sex, n (%)	2,058				0.009 [b]
Male		1,438 (69.9)	1,301 (70.8)	137 (62.3)	
Female		620 (30.1)	537 (29.2)	83 (37.7)	
Witnessed CA, n (%)	2,034				0.071 [b]
Yes		1,902 (93.5)	1,691 (93.2)	211 (96.3)	
No		132 (6.5)	124 (6.8)	8 (3.7)	
Initial rhythm, n (%)	1,717				<0.001 [b]
Shockable		1,242 (72.3)	1,142 (74.3)	100 (55.6)	

For peer review only - http://bmjopen.bmj.com/site/about/guidelines.xhtml

Non-shockable		475 (27.7)	395 (25.7)	80 (44.4)	
Time to defibrillation (min), Mdn (q1-q3)	1,173	2 (1-8)	2 (1-8)	2 (1-7)	0.544 [a]
Place, n (%)	2,058				0.011 [b]
IHCA		1,309 (63.6)	1,152 (62.7)	157 (71.4)	
OHCA		749 (36.4)	686 (37.3)	63 (28.6)	
Defibrillation, n (%)	2,031				0.002 [b]
Yes		1,297 (63.9)	1,181 (65.0)	116 (54.2)	
No		734 (36.1)	636 (35.0)	98 (45.8)	
Etiology, n (%)	2,026				
Heart disease		1,472 (72.7)	1,339 (74.1)	133 (61.0)	<0.001 [b]
Other		554 (27.3)	469 (25.9)	85 (39.0)	

CPC=Cerebral Performance Category, IHCA=In Hospital Cardiac Arrest, OHCA=Out of Hospital Cardiac Arrest

[a] Mann-Whitney U test

[b] Chi-Square test

For peer review only

For peer review only - http://bmjopen.bmj.com/site/about/guidelines.xhtml

Table 2. Differences in self-reported health between survivors with good cerebral performance (CPC 1) and survivors with moderate cerebral disability (CPC 2).

		All n=2,058	CPC 1 n=1,838	CPC 2 n=220	p-value [b]
EQ-5D-5L	Mobility, Mdn (q1-q3)[a]	1 (1-2)	1 (1-2)	2 (1-3)	<0.001
	Self-care, Mdn (q1-q3)[a]	1 (1-1)	1 (1-1)	1 (1-2)	<0.001
	Usual activities, Mdn (q1-q3)	1 (1-2)	1 (1-2)	3 (2-4)	<0.001
	Pain/discomfort, Mdn (q1-q3)	2 (1-3)	2 (1-2)	2 (1-3)	<0.001
	Anxiety/depression, Mdn (q1-q3)[a]	1 (1-2)	1 (1-2)	2 (1-3)	<0.001
EQ VAS	EQ VAS, Mdn (q1-q3)	75 (60-85)	75 (60-85)	60 (45-70)	<0.001
HADS	Anxiety, Mdn (q1-q3)	2 (0-5)	2 (0-5)	4 (1-7)	<0.001
	Depression, Mdn (q1-q3)	2 (1-5)	2 (0-4)	5 (3-8)	<0.001

For peer review only - http://bmjopen.bmj.com/site/about/guidelines.xhtml

CPC=Cerebral Performance Category, EQ-5D-5L=EQ-5D descriptive system five-levels, EQ VAS=EQ Visual Analogue Scale, HADS=Hospital Anxiety and Depression Scale

[a] Based on 2,057 observations

[b] Mann-Whitney U test

For peer review only

For peer review only - http://bmjopen.bmj.com/site/about/guidelines.xhtml

Table 3. Associations between cerebral function (CPC) and self-reported health (EQ VAS and HADS); multiple linear regression analyses adjusted for age, sex, place of cardiac arrest, etiology, and initial rhythm (n=2,025).

Outcome variables	Explanatory variables	B (SE)	95% CI for B	p-value
EQ VAS	CPC 2	-15.02 (1.39)	-17.75 / -12.29	<0.001
	Model statistics	$F(7, 2{,}017)=31.84$, $p<0.001$, $R^2=0.10$		
HADS anxiety	CPC 2	1.86 (0.27)	1.33 / 2.38	<0.001
	Model statistics	$F(7, 2{,}017)=23.3$, $p<0.001$, $R^2=0.08$		
HADS depression	CPC 2	2.61 (0.25)	2.12 / 3.12	<0.001
	Model statistics	$F(7, 2{,}017)=20.7$, $p<0.001$, $R^2=0.07$		

The adjusting co-variates are excluded from the table.

CPC=Cerebral Performance Category, EQ VAS=EQ Visual Analogue Scale, HADS=Hospital Anxiety and Depression Scale, IHCA=In Hospital Cardiac Arrest, OHCA=Out of Hospital Cardiac Arrest

For peer review only - http://bmjopen.bmj.com/site/about/guidelines.xhtml

For peer review only

Table 4. Differences between survivors with good cerebral performance (CPC 1) and

For peer review only - http://bmjopen.bmj.com/site/about/guidelines.xhtml

survivors with moderate cerebral disability (CPC 2) in HADS cut-off score.

	All<br>n=2,058	CPC 1<br>n=1,838	CPC 2<br>n=220	p-value
Anxiety, n (%)				<0.001
Normal range (0-7)	1,778 (86.4)	1,611 (87.6)	167 (75.9)	
Suggested presence (8-10)	165 (8.0)	140 (7.6)	25 (11.4)	
Probable presence (11-21)	115 (5.6)	87 (4.7)	28 (12.7)	
Depression, n (%)				<0.001
Normal range (0-7)	1,797 (87.3)	1,641 (89.3)	156 (70.9)	
Suggested presence (8-10)	159 (7.7)	117 (6.4)	42 (19.1)	
Probable presence (11-21)	102 (5.0)	80 (4.4)	22 (10.0)	

HADS=Hospital Anxiety and Depression Scale, CPC=Cerebral Performance Category

For peer review only

For peer review only - http://bmjopen.bmj.com/site/about/guidelines.xhtml

Table 5. Association between cerebral function (CPC) and self-reported health (EQ-5D-5L); multiple ordinal logistic regression analyses adjusted for age, sex, place of cardiac arrest, etiology, and initial rhythm (n=2,024-2,025).

Outcome variables	Explanatory variables	OR (SE)	95% CI for OR	p-value
Mobility	CPC 2	3.75 (0.50)	2.89 / 4.86	<0.001
	Model statistics	LR χ^2(7, n=2,024)=350.7, p<0.001, McFadden R^2=0.08		
Self-care	CPC 2	4.30 (0.64)	3.21 / 5.77	<0.001
	Model statistics	LR χ^2(7, n=2,024)=202.8, p<0.001, McFadden R^2=0.08		
Usual activities	CPC 2	4.88 (0.65)	3.76 / 6.33	<0.001
	Model statistics	LR χ^2(7, n=2,025)=222.7, p<0.001, McFadden R^2=0.05		
Pain/Discomfort	CPC 2	2.00 (0.27)	1.54 / 2.60	<0.001
	Model statistics	LR χ^2(7, n=2,025)=107.85, p<0.001, McFadden R^2=0.02		

For peer review only - http://bmjopen.bmj.com/site/about/guidelines.xhtml

Anxiety/Depression	CPC 2	3.02 (0.42)	2.31 / 3.96	<0.001
	Model statistics	LR $\chi^2(7, n=2{,}024)=144.69$, $p<0.001$, McFadden $R^2=0.03$		

The adjusting co-variates are excluded from the table.

CPC=Cerebral Performance Category, EQ-5D-5L=EQ-5D descriptive system five-levels, IHCA=In Hospital Cardiac Arrest, OHCA=Out of Hospital Cardiac Arrest

For peer review only

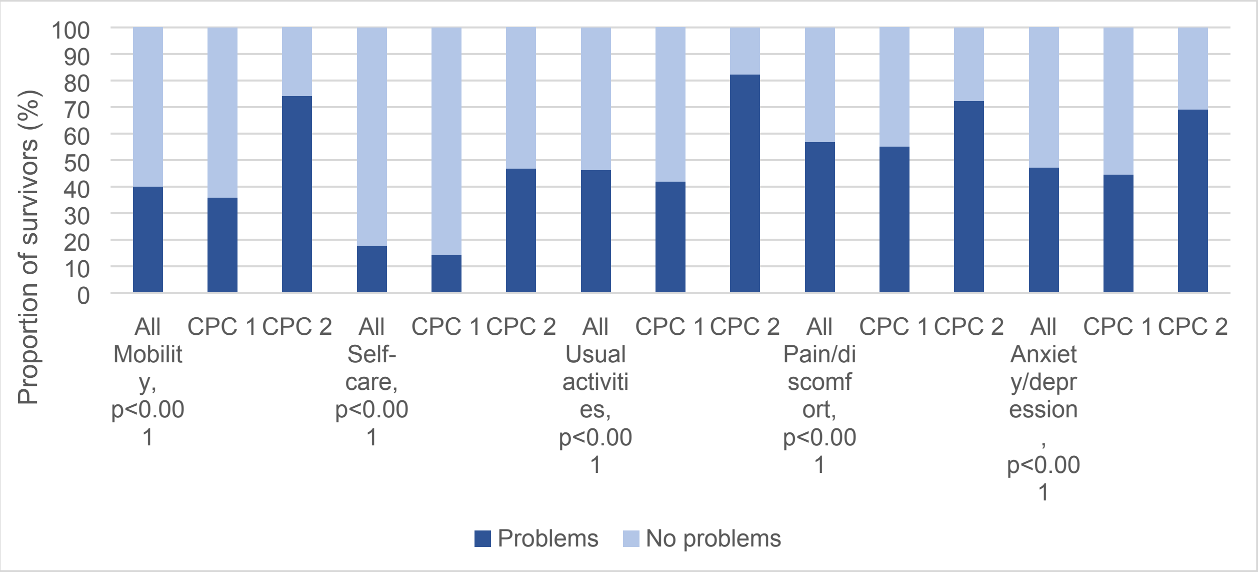

Figure 1. Prevalence scoring of the EQ-5D-5L health dimensions, dichotomized into no problems (1) and problems (2-5), in relation to neurological outcome (good cerebral performance = CPC 1 and moderated cerebral disability = CPC 2).

For peer review only - http://bmjopen.bmj.com/site/about/guidelines.xhtml

STROBE Statement—Checklist of items that should be included in reports of ***cross-sectional studies***

	Item No	Recommendation	Page No
**Title and abstract**	1	(*a*) Indicate the study's design with a commonly used term in the title or the abstract	1
		(*b*) Provide in the abstract an informative and balanced summary of what was done and what was found	2
**Introduction**			
Background/rationale	2	Explain the scientific background and rationale for the investigation being reported	4-5
Objectives	3	State specific objectives, including any prespecified hypotheses	5
**Methods**			
Study design	4	Present key elements of study design early in the paper	5
Setting	5	Describe the setting, locations, and relevant dates, including periods of recruitment, exposure, follow-up, and data collection	5-6
Participants	6	(*a*) Give the eligibility criteria, and the sources and methods of selection of participants	6
Variables	7	Clearly define all outcomes, exposures, predictors, potential confounders, and effect modifiers. Give diagnostic criteria, if applicable	6-7
Data sources/ measurement	8*	For each variable of interest, give sources of data and details of methods of assessment (measurement). Describe comparability of assessment methods if there is more than one group	*N/A*
Bias	9	Describe any efforts to address potential sources of bias	8
Study size	10	Explain how the study size was arrived at	N/A
Quantitative variables	11	Explain how quantitative variables were handled in the analyses. If applicable, describe which groupings were chosen and why	7-8
Statistical methods	12	(*a*) Describe all statistical methods, including those used to control for confounding	7-8
		(*b*) Describe any methods used to examine subgroups and interactions	7-8
		(*c*) Explain how missing data were addressed	N/A
		(*d*) If applicable, describe analytical methods taking account of sampling strategy	N/A
		(*e*) Describe any sensitivity analyses	N/A
**Results**			
Participants	13*	(a) Report numbers of individuals at each stage of study—eg numbers potentially eligible, examined for eligibility, confirmed eligible, included in the study, completing follow-up, and analysed	8
		(b) Give reasons for non-participation at each stage	6
		(c) Consider use of a flow diagram	N/A
Descriptive data	14*	(a) Give characteristics of study participants (eg demographic, clinical, social) and information on exposures and potential confounders	Table 1.
		(b) Indicate number of participants with missing data for each variable of interest	Table 1-5
Outcome data	15*	Report numbers of outcome events or summary measures	9-10

For peer review only - http://bmjopen.bmj.com/site/about/guidelines.xhtml

Main results	16	(*a*) Give unadjusted estimates and, if applicable, confounder-adjusted estimates and their precision (eg, 95% confidence interval). Make clear which confounders were adjusted for and why they were included.	Table 3,5 and p 8,9,10
		(*b*) Report category boundaries when continuous variables were categorized	Figure 1, p 7
		(*c*) If relevant, consider translating estimates of relative risk into absolute risk for a meaningful time period	N/A
Other analyses	17	Report other analyses done—eg analyses of subgroups and interactions, and sensitivity analyses	6
**Discussion**			
Key results	18	Summarise key results with reference to study objectives	11-13
Limitations	19	Discuss limitations of the study, taking into account sources of potential bias or imprecision. Discuss both direction and magnitude of any potential bias	13-14
Interpretation	20	Give a cautious overall interpretation of results considering objectives, limitations, multiplicity of analyses, results from similar studies, and other relevant evidence	14
Generalisability	21	Discuss the generalisability (external validity) of the study results	13
**Other information**			
Funding	22	Give the source of funding and the role of the funders for the present study and, if applicable, for the original study on which the present article is based	15

*Give information separately for exposed and unexposed groups.

Note: An Explanation and Elaboration article discusses each checklist item and gives methodological background and published examples of transparent reporting. The STROBE checklist is best used in conjunction with this article (freely available on the Web sites of PLoS Medicine at http://www.plosmedicine.org/, Annals of Internal Medicine at http://www.annals.org/, and Epidemiology at http://www.epidem.com/). Information on the STROBE Initiative is available at www.strobe-statement.org.

For peer review only

BMJ Open

Differences in self-reported health between cardiac arrest survivors with good cerebral performance and survivors with moderate cerebral disability – a nationwide register study

Journal:	*BMJ Open*
Manuscript ID	bmjopen-2021-058945.R1
Article Type:	Original research
Date Submitted by the Author:	24-May-2022
Complete List of Authors:	Larsson, Karin; Linkoping University Department of Health Medicine and Caring Sciences,<br>Hjelm, Carina; Linkoping University Department of Health Medicine and Caring Sciences<br>Lilja, Gisela ; Lund University, Department of Clinical Sciences<br>Stromberg, Anna; Linkoping University Department of Health Medicine and Caring Sciences; Department of cardiology, Linkoping University<br>Årestedt, Kristofer; Linnaeus University Faculty of Health and Life Sciences; The Research Section, Region Kalmar County
**Primary Subject Heading**:	Cardiovascular medicine
Secondary Subject Heading:	Intensive care, Nursing
Keywords:	Adult cardiology < CARDIOLOGY, Coronary heart disease < CARDIOLOGY, Rehabilitation medicine < INTERNAL MEDICINE, INTENSIVE & CRITICAL CARE

SCHOLARONE™
Manuscripts

I, the Submitting Author has the right to grant and does grant on behalf of all authors of the Work (as defined in the below author licence), an exclusive licence and/or a non-exclusive licence for contributions from authors who are: i) UK Crown employees; ii) where BMJ has agreed a CC-BY licence shall apply, and/or iii) in accordance with the terms applicable for US Federal Government officers or employees acting as part of their official duties; on a worldwide, perpetual, irrevocable, royalty-free basis to BMJ Publishing Group Ltd ("BMJ") its licensees and where the relevant Journal is co-owned by BMJ to the co-owners of the Journal, to publish the Work in this journal and any other BMJ products and to exploit all rights, as set out in our licence.

The Submitting Author accepts and understands that any supply made under these terms is made by BMJ to the Submitting Author unless you are acting as an employee on behalf of your employer or a postgraduate student of an affiliated institution which is paying any applicable article publishing charge ("APC") for Open Access articles. Where the Submitting Author wishes to make the Work available on an Open Access basis (and intends to pay the relevant APC), the terms of reuse of such Open Access shall be governed by a Creative Commons licence – details of these licences and which Creative Commons licence will apply to this Work are set out in our licence referred to above.

Other than as permitted in any relevant BMJ Author's Self Archiving Policies, I confirm this Work has not been accepted for publication elsewhere, is not being considered for publication elsewhere and does not duplicate material already published. I confirm all authors consent to publication of this Work and authorise the granting of this licence.

For peer review only

Differences in self-reported health between cardiac arrest survivors with good cerebral performance and survivors with moderate cerebral disability – a nationwide register study

Karin Larsson RN PhD student[1], Carina Hjelm RN PhD[1], Gisela Lilja OT PhD[2], Anna Strömberg RN Professor[1,3], Kristofer Årestedt RN Professor[4,5]

1 Department of Health, Medicine and Caring Sciences, Linköping University, SE-581 83, Linköping, Sweden

2 Lund University, Skane University Hospital, Department of Clinical Sciences Lund, Neurology, SE-221 85, Lund, Sweden

3 Department of Cardiology, Linköping University, SE-581 83, Linköping, Sweden

4 Faculty of Health and Life Sciences, Linnaeus University, SE-391 82, Kalmar, Sweden

5 The Research Section, Region Kalmar County, SE-391 26, Kalmar, Sweden

Corresponding author: Karin Larsson, RN, PhD student

Department of Health, Medicine and Caring Sciences, Linköping University

SE- 581 83, Linköping

Sweden

Email: karin.larsson@liu.se

Phone: +46 13 284606

Word count Paper: 2933, Word count Abstract: 218

Figures: 1

Tables: 5

For peer review only

ABSTRACT

Objective: The aim was to compare self-reported health of cardiac arrest survivors with good cerebral performance (CPC 1) and survivors with moderate cerebral disability (CPC 2).

Methods: This comparative register study was based on nationwide data from the Swedish Register of Cardiopulmonary Resuscitation. The study included 2,058 in-hospital and out-of-hospital cardiac arrest survivors with good cerebral performance or survivors with moderate cerebral disability, 3-6 months post cardiac arrest. Survivors completed a questionnaire including the Hospital Anxiety and Depression Scale (HADS) and EQ-5D five-levels (EQ-5D-5L). Data were analysed using ordinal and linear regression models.

Results: For all survivors, the prevalence of anxiety and depression symptoms measured by the HADS was 14% and 13% respectively. Using the EQ-5D-5L, the cardiac arrest survivors reported most health problems relating to pain/discomfort (57%), followed by anxiety/depression (47%), usual activities (46%), mobility (40%), and self-care (18%). Compared to the survivors with good cerebral performance, survivors with moderate cerebral disability reported significantly higher symptom levels of anxiety and depression measured with HADS, and poorer health in all dimensions of the EQ-5D-5L after adjusting for age, sex, place of cardiac arrest, aetiology, and initial rhythm ($p<0.001$).

Conclusions: These findings stress the importance of screening for health problems in all cardiac arrest survivors to identify those in need of professional support and rehabilitation, independent on neurological outcome.

Keywords: Anxiety, Cerebral performance, Depression, Health status, Heart arrest, Self-reported health

For peer review only - http://bmjopen.bmj.com/site/about/guidelines.xhtml

Strengths and limitations of this study:

- The study includes a large sample of cardiac arrest survivors (n=2,058), both hospital and out-of-hospital cardiac arrest survivors with a favourable neurological outcome.
- The data is taken from the Swedish Register of Cardiopulmonary Resuscitation, a nationwide registry with nearly complete coverage of all cardiac arrest in Sweden where cardiopulmonary resuscitation is started.
- The cross-sectional design omits any causal conclusions.
- The reason for dropouts is un-known and non-response bias can therefore not be excluded.

For peer review only

INTRODUCTION

Cardiac arrest (CA) is a global health problem with poor survival. The survival rate varies across studies and countries but is, according to recent studies, around 20% for in-hospital cardiac arrest (IHCA)[1] and 10% for out-of-hospital cardiac arrest (OHCA).[2] CA results rapidly in hypoxic-ischaemic brain damage[3] and the neurological outcome is therefore an important outcome, commonly assessed with the Cerebral Performance Category (CPC) scale.[4] The CPC classification is made by health care professionals on a scale ranging from 1 to 5, where CPC 1 and 2 (i.e., good cerebral performance and moderate cerebral disability) are generally defined as a favourable neurological outcome.[5-8] In contrast CPC 3 to 5 (severe cerebral disability to brain death) are defined as unfavourable neurological outcomes and imply dependency on others.[4,9]

Health-related quality of life (HRQoL) reflects the outcome after a CA from the survivor's perspective and has been recommended as one of the core outcomes post CA.[10] Health is a broad concept defined by WHO as "a state of complete physical, mental and social well-being and not merely the absence of disease or infirmity".[11] Previous studies have shown that CA survivors in general report good health.[12-14] In fact, one comparative study showed that survivors reported even higher levels of health compared to a sex- and age-matched normal population.[14] However, a significant minority of the CA survivors report severe health problems, particularly with symptoms of anxiety and depression.[12-13,15]

Although cerebral performance and self-reported health are both important outcomes, the association between them is not well explored among CA survivors. A study by Geri et al.[16] found that survivors with good cerebral performance (CPC 1) reported better health compared to survivors with moderate or severe cerebral disability (CPC 2 and 3). No study has been

For peer review only

identified that has specifically investigated differences in self-reported health of CA survivors with good cerebral performance (CPC 1) and moderate cerebral disability (CPC 2). As these survivors are defined as having a favourable neurological outcome, there is a risk that health problems could be overlooked. Therefore, the aim of the present study was to compare self-reported health between cardiac arrest survivors with good cerebral performance (CPC 1) and survivors with moderate cerebral disability (CPC 2).

METHODS

Design

This comparative registry study was based on data from the Swedish Register of Cardiopulmonary Resuscitation. Overall, the data is the same as was used in a study by Djärv et al.[15] that focused on the differences in HRQoL between IHCA and OHCA survivors. However, the present study does not include survivors with CPC >2.

Setting

The Swedish Register of Cardiopulmonary Resuscitation (https://shlrsjh.registercentrum.se/) is a nationwide quality register consisting of two parts: IHCA and OHCA. All cardiac arrests are registered when cardiopulmonary resuscitation (CPR) has been started. The CA survivors are informed of the enrolment in the registry and can withdraw their consent at any time. The register covers almost 100% of OHCA and a majority of IHCA in Sweden. All survivors 18 years or older who are still alive 3–6 months post CA are screened for eligibility to complete a questionnaire about self-reported health by resuscitation coordinators or cardiac rehabilitation nurses. Unwillingness to participate, severe cognitive dysfunction, language difficulties, and severe physical and/or psychological difficulties (according to the latest medical file) are criteria for exclusion in the register during the study period. A letter is sent to the CA

For peer review only - http://bmjopen.bmj.com/site/about/guidelines.xhtml

For peer review only

survivors, including information about the registry, two questionnaires (the Hospital Anxiety and Depression Scale and EQ-5D-5L), and an invitation for a structured interview by phone. The CA survivors are instructed to complete the questionnaires during the structured interview, which also contain an assessment of cerebral performance measured by the CPC score. The interview is performed by the resuscitation coordinators or cardiac rehabilitation nurses. A short-written manual is followed to standardize the assessment of the CPC score.

Sample and procedure

The present study included registry data collected between 1st of January 2014 and 31st of December 2017. During this period, 2,141 CA survivors, 772 OHCA (response rate 54.6%) and 1,369 IHCA (response rate 57.4%), had completed the questionnaire. Of these, 57 survivors had CPC ≥3 and 26 (1.2%) had missing data on the CPC assessment and were therefore excluded. Thus, the final sample consisted of 2,058 survivors.

Patient and public involvement

The study is based on the Swedish Register of Cardiopulmonary Resuscitation; neither patients nor the public were involved. Ethical approval is needed to use registry data.

Outcome measures of self-reported health

Symptoms of anxiety and depression

The Hospital Anxiety and Depression Scale (HADS) is developed to measure symptoms of anxiety and depression during the last week. The instrument consists of two sub-scales, HADS anxiety and HADS depression. Each subscale includes seven items, which all have four response options (0-3). The responses to the items in each subscale are summed to a subscale score that can vary between 0 and 21; higher scores indicate higher symptom

For peer review only - http://bmjopen.bmj.com/site/about/guidelines.xhtml

level.[17] The following cut-off values have been recommended: 0-7 normal range, 8-10 suggested presence of anxiety and/or depression and 11-21 probable presence of anxiety and/or depression.[18] Both subscale scores and cut-off scores were used in the present study. The instrument has previously been used in CA research[12,19] and has shown good measurement properties.[20] Cronbach's alpha was 0.88 and 0.85 for the anxiety and depression subscales respectively in the present study.

Health status

The EQ-5D-5L is a generic instrument to measure health status and includes three parts: EQ-5D-5L descriptive system, EQ VAS and EQ-5D value index (the latter not used in the present study).[21] The EQ-5D-5L descriptive system consists of five health dimensions: mobility, self-care, usual activities, pain/discomfort, and anxiety/depression. Each dimension has five response options, referred to as levels, ranging from no problems (1) unable to do/extreme problems (5). These scores reflect the intensity of the health problems. The five levels can also be dichotomised into no problem (1) and problems (2-5), reflecting the prevalence of the health problems. Both prevalence and intensity scoring are used in the present study. The EQ VAS is a measure of overall health and graded on a 0-100 scale, where 0 represents worst possible health and 100 best possible health.[21] The EQ-5D-5L has been recommended as a core outcome in CA research[10] and has in previous studies shown good measurement properties in different patient groups, e.g. stroke and chronic obstructive pulmonary disease.[22-23]

Statistical analysis

To present the characteristics of the survivors and the variables, descriptive statistics were used. To compare self-reported health between CA survivors with good cerebral performance

For peer review only - http://bmjopen.bmj.com/site/about/guidelines.xhtml

For peer review only

(CPC 1) and survivors with moderate cerebral disability (CPC 2), chi-square test was used for nominal data and Mann-Whitney U test was used for ordinal data.

Multiple linear regression analyses were used to investigate associations between cerebral function (CPC as the explanatory variable) and self-reported health (the EQ VAS and HADS as outcome variables). Multiple ordinal logistic regression analyses were used to handle the ordinal nature of data in the EQ-5D-5L health dimensions. Cerebral function (CPC 1=0, CPC 2=1) was included as an explanatory variable and the EQ-5D-5L health dimensions as outcome variables. Both the linear and ordered logistic regression models were adjusted for covariates that are commonly used in cardiac arrest research;[12,15] age, sex (male=0, female=1), place of CA (IHCA=0, OHCA=1), aetiology (heart disease=1, other=0), and initial rhythm. Initial rhythm was coded as shockable rhythm (VF/VT) and non-shockable rhythm (PEA and asystole). To handle the large number of missing data in initial rhythm (n=341), they were coded within a third 'unknown' group. Thus, initial rhythm was included as a dummy coded variable in the regression models, with shockable rhythm as a reference category.

The level of statistical significance was set at $p<0.05$ and the data were analysed using SPSS statistics 24 (IBM Corp., Armonk, NY, USA) and Stata 16.1 (StataCorp LLC, College Station, TX, USA).

RESULTS

Survivor characteristics

The final sample consisted of 2,058 survivors: 1,309 IHCA survivors and 749 OHCA survivors. The median age was 68 years (range=59-75), and the majority were male (n=1,438,

For peer review only - http://bmjopen.bmj.com/site/about/guidelines.xhtml

70%). Most survivors were reported to have good cerebral performance (CPC 1, n=1,838, 89%). A majority of the cardiac arrests were witnessed (n=1,902, 94%) and caused by heart disease (n=1,472, 73%). About three out of four survivors had an initial shockable rhythm (n=1,242, 72%), and 63% (n=1,297) were defibrillated. The median time to defibrillation was 2 minutes (q1-q3=1-8), 1 minute for IHCA and 8 minutes for OHCA. Compared to survivors with moderate cerebral disability, those with good cerebral performance were significantly younger ($p=0.002$), more often male ($p=0.009$), had more often an initial shockable rhythm ($p<0.001$), were more often defibrillated ($p=0.002$) and more often had heart disease as the cause of CA ($p<0.001$). In addition, the share of OHCA ($p=0.011$) was larger in the group of survivors with good cerebral performance than in the group of survivors with moderate cerebral disability. The difference in time to defibrillation and whether the CA was witnessed did not show any significant difference between survivors with good cerebral performance and survivors with moderate cerebral disability (Table 1).

Symptoms of anxiety and depression (HADS)

Subscale-scores for the HADS showed that the survivors generally reported low symptom levels of anxiety (Mdn=2, q1-q3=0-5) and depression (Mdn=2, q1-q3=1-5). However, survivors with moderate cerebral disability reported significantly higher symptom levels of both anxiety (ΔMdn=2, $p<0.001$) and depression (ΔMdn=3, $p<0.001$) than survivors with good cerebral performance (Table 2). Based on the multiple linear regression analyses, these differences remained when age, sex, place of CA, aetiology, and initial rhythm were adjusted for ($p<0.001$). The regression model explained 8% of the total variance in symptoms of anxiety and 7% in symptoms of depression (Table 3).

For peer review only - http://bmjopen.bmj.com/site/about/guidelines.xhtml

Using the cut-off scores for the HADS, 280 (14%) of all the CA survivors reported having suggested or probable presence of anxiety and 261 (13%) suggested or probable presence of depression. However, the share of survivors with suggested or probable presence of anxiety and depression was significantly higher among survivors with moderate cerebral disability than in survivors with good cerebral performance ($p<0.001$). Among the survivors with moderate cerebral disability, 53 (24%) reported having symptoms of anxiety and 64 (29%) symptoms of depression (Table 4).

Health status (EQ-5D-5L and EQ VAS)

Using the EQ-5D-5L descriptive systems, the CA survivors reported pain/discomfort as the most prevalent health problem (n=1172, 56.9%), followed by anxiety/depression (n=971, 47.2%), usual activities (n=952, 46.3%), mobility (n=822, 40.0%) and self-care (n=365, 17.7%). All health problems were significantly more prevalent among survivors with moderate cerebral disability than survivors with good cerebral performance ($p<0.001$) (Fig.1).

Although health problems were common, the CA survivors reported, on average, low intensity levels of all health problems (Mdn=1-2). However, the survivors with moderate cerebral disability reported significantly higher intensity levels for all health dimensions compared to the survivors with good cerebral performance ($p<0.001$). Survivors with good cerebral performance reported the highest levels of pain/discomfort (Mdn=2, q1-q3=1-2) while survivors with moderate cerebral disability reported the highest levels of usual activities (Mdn=3, q1-q3=2-4) (Table 2). Based on the ordinal regression analyses, these group differences remained when age, sex, place of CA, aetiology, and initial rhythm were included as adjusting covariates ($p<0.001$). The pseudo R^2 values (McFadden) ranged between 0.02 and 0.08 (Table 5).

For peer review only - http://bmjopen.bmj.com/site/about/guidelines.xhtml

The CA survivors reported overall a median score of 75 (q1-q3= 60-85) on the EQ VAS; survivors with moderate cerebral disability reported significantly worse health status than survivors with good cerebral performance (ΔMdn=15, p<0.001) (Table 2). Based on the multiple linear regression analysis, this group difference remained in the adjusted model (p<0.001). The regression model explained 10% of the total variance in EQ VAS (Table 3).

DISCUSSION

To the best of our knowledge, this is the largest study to have compared self-reported health between CA survivors with good cerebral performance (CPC 1) and survivors with moderate cerebral disability (CPC 2). Although these groups are commonly defined as having a favourable neurological outcome after their CA, the results show that survivors with moderate cerebral disability reported significantly more health problems, measured by the HADS and EQ-5D-5L, compared to survivors with good cerebral performance. These differences remained after adjustment for age, sex, place of CA, aetiology, and initial rhythm.

The survivors generally reported low symptom levels of anxiety and depression, but a great difference was shown between those with moderate cerebral disability and those with good cerebral performance. The prevalence of anxiety and depression in the present study corresponds well with a systematic review about psychological health post CA.[24] Most of the included studies that had used the HADS reported a prevalence of 13-14% of both anxiety and depression, thus levels were similar to those shown for the whole sample in the present study. It should be noted that the prevalence of anxiety and depression in the present study was significantly higher in survivors with moderate cerebral disability. The prevalence of anxiety in this group corresponds with a Swedish study of 278 OHCA survivors treated with

For peer review only

target temperature management, at six-months' follow-up.[25] However, the prevalence of depression in that study was lower compared to the survivors with moderate cerebral disability in the present study. This finding was unexpected as all patients in the temperature study, in contrast to the present study, had received intensive care and thereby experienced a more complicated disease trajectory.

The prevalence of depression was more common than anxiety in survivors with moderate cerebral disability and the opposite situation was shown for survivors with good cerebral performance. Included studies in a systematic review by Wilder Schaaf et al. reported higher prevalence of anxiety than depression, or equal levels, in CA survivors. However, the opposite situation was not reported. It should be noted that only OHCA survivors were included in that review, the number of included studies was small (n=11), and the sample size in all included studies was small (21-168 survivors). In addition, different measures were used to assess symptoms of anxiety and depression, and only four studies used the HADS.[24] The HADS' ability to differentiate between anxiety and depression has been questioned, and a review about the psychometric properties concluded that the subscales therefore should be compared with caution.[26]

Unfortunately, there are no Swedish normative data for symptoms of anxiety and depression measured by the HADS. Compared to normative data for anxiety and depression measured by the HADS in the UK,[27] the prevalence of anxiety was less common while depression was more common in survivors with good cerebral performance. In addition, the prevalence of having suggested or probable presence of anxiety and depression was more common in survivors with moderate cerebral disability compared to normative data from the UK. It should be noted that normative data from the UK may differ from the general population in

For peer review only - http://bmjopen.bmj.com/site/about/guidelines.xhtml

Sweden. However, the results indicate that symptoms of anxiety and depression need to be acknowledged also in CA survivors with favourable neurological outcome.

Survivors with moderate cerebral disability also reported more problems with their physical and overall health, measured with the EQ-5D-5L, compared to survivors with good cerebral performance. Age and comorbidity may explain these differences as survivors with moderate cerebral disability were significantly older and more often suffered ICHA compared to survivors with good cerebral performance. However, the multiple regression analyses in the present study show that age could not explain these differences. Unfortunately, data of comorbidity is not registered for OHCA survivors in the Swedish Register of Cardiopulmonary Resuscitation and could therefore not be examined in the present study. It is likely that comorbidity is an important confounder as previous studies have shown that comorbidity is related to self-reported health in CA survivors[28] and is likely to be related to cerebral performance as well. Comorbidity should therefore be considered in future research.

Compared to a Swedish population-based study, including 25,867 adults between 30-103 years,[29] the CA survivors with good cerebral performance reported equal median scores on the EQ VAS as persons with low education level, the group with poorest health status in the general population. However, survivors with moderate cerebral disability reported in average 15 points lower on the EQ VAS compared to persons with low education level in the general population. Moreover, the presence of health problems in the five health domains in EQ-5D-5L were similar between the survivors with good cerebral performance and the general population. In contrast, survivors with moderate cerebral disability reported more health problems in all health dimensions in EQ-5D-5L than persons with low education in the general population. The CA survivors in the present study also reported lower median scores

For peer review only - http://bmjopen.bmj.com/site/about/guidelines.xhtml

on EQ VAS compared to survivors who had been treated with therapeutic hypothermia six months earlier.[30] Thus, problems with self-reported health needs to be taken seriously also in CA survivors with favourable neurological outcome.

Methodological limitations

The study has some limitations that need to be considered. No causal relationship can be drawn due to the cross-sectional design. The no-response rate was acceptable; however, the reasons for not participating are not known. It is a potential risk that the response rate is lower among survivors with moderate cerebral disability and those with poor self-reported health. In addition, survivors with severe physical and/or psychological difficulties were excluded from the third registration. Therefore, we cannot be sure that the participants are representative of the entire population of CA survivors. Information about survivors' cerebral performance before CA is unknown and therefore, we do not know if the impaired cerebral performance among those survivors who were classified according to CPC 2 was caused by the CA. The use of CPC may be criticized as it did not take to account prior disability. Of this reason, other measures like the Modified Ranking Scale and extended Glasgow Outcome Scale are commonly recommended to be used in cardiac arrest survivors. However, the correlation between these outcome measures is high.[10,31] To exclude the risk that the assessment of the CPC at the follow-up assessment would be affected by the survivors' self-reported health during the interview, the agreement with CPC at hospital discharge was examined. The absolute agreement was 81% and the risk for that is therefore rather small. The HADS and EQ-5D-5L were used to measure self-reported health as they are recommended in international guidelines for post CA care.[10,32] However, neither HADS nor EQ-5D-5L are disease specific for CA survivors and might therefore not capture specific health problems related to surviving a life-threatening condition such as CA. Even so, the instruments capture

For peer review only - http://bmjopen.bmj.com/site/about/guidelines.xhtml

important health problems, and early screening could be a way to identify survivors at risk of low self-reported health, also in survivors with favourable neurological outcome, particularly in survivors with moderate cerebral disability.

CONCLUSIONS

This study contributes with important knowledge about cerebral performance and self-reported health in CA survivors with favourable neurological outcome. Survivors with moderate cerebral disability (CPC 2) reported significantly lower levels of self-reported health compared to survivors with good cerebral performance (CPC 1). These findings stress the importance of screening for health problems in all cardiac arrest survivors to identify those in need of professional support and rehabilitation, independent of neurological outcome. Until there are CA-specific instruments to measure self-reported health, the HADS and EQ-5D-5L serve as adequate screening instruments.

Acknowledgements

We would like to thank all the participating survivors in the Swedish Register of Cardiopulmonary Resuscitation.

Contributors

KL: contributed to the conception of the design, the acquisition of the data, analysis, and interpretation of data, drafting and revising the work critically for important intellectual content and approval of the final manuscript.

CH: contribute to the conception of the design, interpretation of data, drafting and revising the work critically for important intellectual content and approval of the final manuscript.

For peer review only - http://bmjopen.bmj.com/site/about/guidelines.xhtml

GL: contributed to the conception of the design, revising the work critically for important intellectual content and approval of the final manuscript.

AS: contributed to the conception of the design, revising the work critically for important intellectual content and approval of the final manuscript.

KÅ: contributed to the conception of the design, the acquisition of the data, analysis, and interpretation of data, drafting and revising the work critically for important intellectual content and approval of the final manuscript.

Funding

This work was supported by The Medical Research Council of Southeast Sweden (FORSS-745341 and FORSS-939845) and Region Östergötland, Linköping University (RÖ-920281), Sweden.

Competing interests

None to declare.

Patient consent for publication

Not required.

Ethics approval

The study was approved by the Regional Ethical Review Board in Linköping, Sweden (No. 2018/480-21).

Data availability statement

Data is available upon request from the author KL.

For peer review only

For peer review only

For peer review only - http://bmjopen.bmj.com/site/about/guidelines.xhtml

References

1 Andersen LW, Holmberg MJ, Berg KM, et al. In-hospital cardiac arrest: a review. *JAMA Intern Med* 2019;321:1200-10 doi:10.1001/jama.2019.1696 [published Online First: 25 April 2019].

2 Shijiao Y, Yong G, Nan J, et al. The global survival rate among adult out-of-hospital cardiac arrest patients who received cardiopulmonary resuscitation: a systematic review and meta-analysis. *Crit Care* 2020;24:1-13 doi:10.1186/s13054-020-2773-2.

3 Busl KM, Greer DM. Hypoxic-ischemic brain injury: pathophysiology, neuropathology and mechanisms. *NeuroRehabilitation* 2010;26:5-13 doi:10.3233/NRE-2010-0531.

4 Cummins RO, Chamberlain DA, Abramson NS, et al. Recommended guidelines for uniform reporting of data from out-of-hospital cardiac arrest: The Utstein style: a statement for health professionals from a task force of the American Heart Association, the European Resuscitation Council, and Heart and Stroke Foundation of Canada, and the Australian Resuscitation Council. *Circulation* 1991;84:960-75 doi:10.1161/01.CIR.84.2.960.

5 Akin M, Garcheva V, Sieweke J-T, et al. Neuromarkers and neurological outcome in out-of-hospital cardiac arrest patients treated with therapeutic hypothermia–experience from the HAnnover COoling REgistry (HACORE). *PloS one* 2021;16:e0245210 doi:10.1371/journal.pone.0245210.

6 Reynolds EC, Zenasni Z, Harrison DA, et al. How do information sources influence the reported Cerebral Performance Category (CPC) for in-hospital cardiac arrest survivors? An observational study from the UK National Cardiac Arrest Audit (NCAA). *Resuscitation* 2019;114:19-23 doi:10.1016/j.resuscitation.2019.05.035 [published Online First: 12 June 2019].

For peer review only - http://bmjopen.bmj.com/site/about/guidelines.xhtml

7 Eertmans W, Tran TMP, Genbrugge C, et al. A prediction model for good neurological outcome in successfully resuscitated out-of-hospital cardiac arrest patients. *Scand J Trauma Resusc Emerg Med* 2018;26:93 doi:10.1186/s13049-018-0558-2.

8 Gräsner J-T, Herlitz J, Tjelmeland IBM, et al. European Resuscitation Council Guidelines 2021: epidemiology of cardiac arrest in Europe. *Resuscitation* 2021;161:61-79 doi:10.1016/j.resuscitation.2021.02.007 [published Online First: 24 March 2021].

9 Jennett B, Bond M. Assessment of outcome after severe brain damage. A practical scale. *Lancet* 1975;305:480-4 doi:10.1016/S0140-6736(75)92830-5.

10 Haywood K, Whitehead L, Nadkarni VM, et al. COSCA (Core Outcome Set for Cardiac Arrest) in adults: an advisory statement from the International Liaison Committee on Resuscitation. *Cirkulation* 2018;137:e783-e801. doi:10.1161/CIR.0000000000000562 [published Online First: 26 April 2018].

11 WHO. Preamble to the Constitution of WHO as adopted by the International Health Conference, New York, 19 June - 22 July 1946; signed on 22 July 1946 by the representatives of 61 States (Official Records of WHO, no. 2, p. 100) and entered into force on 7 April 1948.

12 Israelsson J, Bremer A, Herlitz J, et al. Health status and psychological distress among in-hospital cardiac arrest survivors in relation to gender. *Resuscitation* 2017;114:27-33 doi:10.1016/j.resuscitation.2017.02.006 [published Online First: 17 February 2017].

13 Moulaert VRM, van Heugten CM, Gorgels TPM, et al. Long-term outcome after survival of a cardiac arrest: a prospective longitudinal cohort study. *Neurorehabil Neural Repair* 2017;31:530-9 doi:10.1177/1545968317697032.

For peer review only

14 Tiainen M, Vaahersalo J, Skrifvars MB, et al. Surviving out-of-hospital cardiac arrest: the neurological and functional outcome and health-related quality of life one year later. *Resuscitation* 2018;129:19-23 doi:10.1016/j.resuscitation.2018.05.011 [published Online First: 15 May 2018].

15 Djärv T, Bremer A, Herlitz J, et al. Health-related quality of life after surviving an out-of-hospital compared to an in-hospital cardiac arrest: A Swedish population-based registry study. *Resuscitation* 2020;151:77-84 doi:10.1016/j.resuscitation.2020.04.002 [published Online First: 12 April 2020].

16 Geri G, Bougouin W, Champigneulle B, et al. Predictors of long-term functional outcome and health-related quality of life after out-of-hospital cardiac arrest. *Resuscitation* 2017;113:77-82 doi:10.1016/j.resuscitation.2017.01.028 [published Online First: 12 February 2017].

17 Zigmond AS, Snaith RP. The hospital anxiety and depression scale. *Acta Psychiatr Scand* 1983;67:361-70 doi:10.1111/j.1600-0447.1983.tb09716.x.

18 Snaith RP. The Hospital Anxiety And Depression Scale. *Health Qual Life Outcomes* 2003;1:29-4 doi:10.1186/1477-7525-1-29.

19 Buanes EA, Flaatten H, Husby T, et al. Cognitive function and health-related quality of life four years after cardiac arrest. *Resuscitation* 2015;89:13-8 doi:10.1016/j.resuscitation.2014.12.021 [published Online First: 14 January 2015].

20 Djukanovic I, Carlsson J, Årestedt K. Is the Hospital Anxiety and Depression Scale (HADS) a valid measure in a general population 65-80 years old? A psychometric evaluation study. *Health Qual Life Outcomes* 2017;15:1-10 doi:10.1186/s12955-017-0759-9.

For peer review only

21 EuroQol. EuroQol - a new facility for the measurement of health-related quality of life. Health policy (Amsterdam, Netherlands) 1990;16:199-208 doi:10.1016/0168-8510(90)90421-9.

22 Nolan CM, Longworth L, Lord J, et al. The EQ-5D-5L health status questionnaire in COPD: validity, responsiveness and minimum important difference. *Thorax* 2016;71: 493-500 doi:10.1136/thoraxjnl-2015-207782 [published Online First: 30 March 2016].

23 Lu W-S, Huang S-L, Yang J-F, et al. Convergent validity and responsiveness of the EQ-5D utility weights for stroke survivors. *J Rehabil Med* 2016;48:346-51 doi: 10.2340/16501977-2069.

24 Wilder Schaaf KP, Artman LK, Peberdy MA, et al. Anxiety, depression, and PTSD following cardiac arrest: a systematic review of the literature. *Resuscitation* 2013;84:873-7 doi:10.1016/j.resuscitation.2012.11.021 [published Online First: 29 November 2012].

25 Lilja G, Nilsson G, Nielsen N, et al. Anxiety and depression among out-of-hospital cardiac arrest survivors. *Resuscitation* 2015;97:68-75 doi:10.1016/j.resuscitation.2015.09.389 [published Online First: 9 October 2015].

26 Cosco TD, Doyle F, Ward M, McGee H. Latent structure of the Hospital Anxiety And Depression Scale: a 10-year systematic review. *J Psychosom Res* 2012;72:180-4 doi:10.1016/j.jpsychores.2011.06.008 [published Online First: 14 September 2011].

27 Breeman S, Cotton S, Fielding S, et al. Normative data for the Hospital Anxiety and Depression Scale. *Qual Life Res* 2015;24:391-8 doi:10.1007/s11136-014-0763-z [published Online First: 27 July 2014].

28 Andrew E, Nehme Z, Bernard S, et al. The influence of comorbidity on survival and long-term outcomes after out-of-hospital cardiac arrest. *Resuscitation* 2017;110:42-7 doi:10.1016/j.resuscitation.2016.10.018 [published Online First: 2 November 2016].

For peer review only - http://bmjopen.bmj.com/site/about/guidelines.xhtml

29 Teni FS, Gerdtham UG, Leidl R, et al. Inequality and heterogeneity in health-related quality of life: findings based on a large sample of cross-sectional EQ-5D-5L data from the Swedish general population. *Qual Life Res* 2022;31:697-712 doi:10.1007/s11136-021-02982-3 [published Online First: 10 October 2021].

30 Larsson IM, Wallin E, Rubertsson S, Kristofferzon ML. Health-related quality of life improves during the first six months after cardiac arrest and hypothermia treatment. *Resuscitation* 2014;85: 215-220 doi:10.1016/j.resuscitation.2013.09.017.

31 Raina KD, Rittenberger JC, Holm MB, et al. Functional outcomes: one year after a cardiac arrest. *Biomed Res Int* 2015 doi:10.1155/2015/283608 [published Online First: 3 September 2015].

32 Nolan JP, Sandroni C, Böttiger BW, et al. European Resuscitation Council and European Society of Intensive Care Medicine Guidelines 2021: post-resuscitation care. *Resuscitation* 2021;161:220-69 doi:10.1007/s00134-021-06368-4 [published Online First: 25 March 2021].

For peer review only

For peer review only

For peer review only - http://bmjopen.bmj.com/site/about/guidelines.xhtml

Table 1. Background data of the survivors with good cerebral performance (CPC 1) and survivors with moderate cerebral disability (CPC 2).

	Valid n	All n=2,058	CPC 1 n=1,838	CPC 2 n=220	p-value
Age (years), Mdn (q1-q3)	2,057	68 (59-75)	68 (58-75)	71 (62-79)	0.002 [a]
Sex, n (%)	2,058				0.009 [b]
Male		1,438 (69.9)	1,301 (70.8)	137 (62.3)	
Female		620 (30.1)	537 (29.2)	83 (37.7)	
Witnessed CA, n (%)	2,034				0.071 [b]
Yes		1,902 (93.5)	1,691 (93.2)	211 (96.3)	
No		132 (6.5)	124 (6.8)	8 (3.7)	
Initial rhythm, n (%)	1,717				<0.001 [b]
Shockable		1,242 (72.3)	1,142 (74.3)	100 (55.6)	

For peer review only - http://bmjopen.bmj.com/site/about/guidelines.xhtml

Non-shockable		475 (27.7)	395 (25.7)	80 (44.4)	
Time to defibrillation (min), Mdn (q1-q3)	1,173	2 (1-8)	2 (1-8)	2 (1-7)	0.544 [a]
Place, n (%)	2,058				0.011 [b]
IHCA		1,309 (63.6)	1,152 (62.7)	157 (71.4)	
OHCA		749 (36.4)	686 (37.3)	63 (28.6)	
Defibrillation, n (%)	2,031				0.002 [b]
Yes		1,297 (63.9)	1,181 (65.0)	116 (54.2)	
No		734 (36.1)	636 (35.0)	98 (45.8)	
Etiology, n (%)	2,026				
Heart disease		1,472 (72.7)	1,339 (74.1)	133 (61.0)	<0.001 [b]
Other		554 (27.3)	469 (25.9)	85 (39.0)	

CPC=Cerebral Performance Category, IHCA=In Hospital Cardiac Arrest, OHCA=Out of Hospital Cardiac Arrest

[a] Mann-Whitney U test

[b] Chi-Square test

For peer review only

For peer review only - http://bmjopen.bmj.com/site/about/guidelines.xhtml

Table 2. Differences in self-reported health between survivors with good cerebral performance (CPC 1) and survivors with moderate cerebral disability (CPC 2).

		All n=2,058	CPC 1 n=1,838	CPC 2 n=220	p-value [b]
EQ-5D-5L	Mobility, Mdn (q1-q3)[a]	1 (1-2)	1 (1-2)	2 (1-3)	<0.001
	Self-care, Mdn (q1-q3)[a]	1 (1-1)	1 (1-1)	1 (1-2)	<0.001
	Usual activities, Mdn (q1-q3)	1 (1-2)	1 (1-2)	3 (2-4)	<0.001
	Pain/discomfort, Mdn (q1-q3)	2 (1-3)	2 (1-2)	2 (1-3)	<0.001
	Anxiety/depression, Mdn (q1-q3)[a]	1 (1-2)	1 (1-2)	2 (1-3)	<0.001
EQ VAS	EQ VAS, Mdn (q1-q3)	75 (60-85)	75 (60-85)	60 (45-70)	<0.001
HADS	Anxiety, Mdn (q1-q3)	2 (0-5)	2 (0-5)	4 (1-7)	<0.001
	Depression, Mdn (q1-q3)	2 (1-5)	2 (0-4)	5 (3-8)	<0.001

For peer review only - http://bmjopen.bmj.com/site/about/guidelines.xhtml

CPC=Cerebral Performance Category, EQ-5D-5L=EQ-5D descriptive system five-levels, EQ VAS=EQ Visual Analogue Scale, HADS=Hospital Anxiety and Depression Scale

[a] Based on 2,057 observations

[b] Mann-Whitney U test

For peer review only

For peer review only - http://bmjopen.bmj.com/site/about/guidelines.xhtml

Table 3. Associations between cerebral function (CPC) and self-reported health (EQ VAS and HADS); multiple linear regression analyses adjusted for age, sex, place of cardiac arrest, etiology, and initial rhythm (n=2,025).

Outcome variables	Explanatory variables	B (SE)	95% CI for B	p-value
EQ VAS	CPC 2	-15.02 (1.39)	-17.75 / -12.29	<0.001
	Model statistics	$F(7, 2{,}017)=31.84$, $p<0.001$, $R^2=0.10$		
HADS anxiety	CPC 2	1.86 (0.27)	1.33 / 2.38	<0.001
	Model statistics	$F(7, 2{,}017)=23.3$, $p<0.001$, $R^2=0.08$		
HADS depression	CPC 2	2.61 (0.25)	2.12 / 3.12	<0.001
	Model statistics	$F(7, 2{,}017)=20.7$, $p<0.001$, $R^2=0.07$		

The adjusting co-variates are excluded from the table.

CPC=Cerebral Performance Category, EQ VAS=EQ Visual Analogue Scale, HADS=Hospital Anxiety and Depression Scale, IHCA=In Hospital Cardiac Arrest, OHCA=Out of Hospital Cardiac Arrest

For peer review only

For peer review only

Table 4. Differences between survivors with good cerebral performance (CPC 1) and

For peer review only - http://bmjopen.bmj.com/site/about/guidelines.xhtml

survivors with moderate cerebral disability (CPC 2) in HADS cut-off score.

	All n=2,058	CPC 1 n=1,838	CPC 2 n=220	p-value
Anxiety, n (%)				<0.001
Normal range (0-7)	1,778 (86.4)	1,611 (87.6)	167 (75.9)	
Suggested presence (8-10)	165 (8.0)	140 (7.6)	25 (11.4)	
Probable presence (11-21)	115 (5.6)	87 (4.7)	28 (12.7)	
Depression, n (%)				<0.001
Normal range (0-7)	1,797 (87.3)	1,641 (89.3)	156 (70.9)	
Suggested presence (8-10)	159 (7.7)	117 (6.4)	42 (19.1)	
Probable presence (11-21)	102 (5.0)	80 (4.4)	22 (10.0)	

HADS=Hospital Anxiety and Depression Scale, CPC=Cerebral Performance Category

For peer review only

Table 5. Association between cerebral function (CPC) and self-reported health (EQ-5D-5L); multiple ordinal logistic regression analyses adjusted for age, sex, place of cardiac arrest, etiology, and initial rhythm (n=2,024-2,025).

Outcome variables	Explanatory variables	OR (SE)	95% CI for OR	p-value
Mobility	CPC 2	3.75 (0.50)	2.89 / 4.86	<0.001
	Model statistics	LR χ^2(7, n=2,024)=350.7, p<0.001, McFadden R^2=0.08		
Self-care	CPC 2	4.30 (0.64)	3.21 / 5.77	<0.001
	Model statistics	LR χ^2(7, n=2,024)=202.8, p<0.001, McFadden R^2=0.08		
Usual activities	CPC 2	4.88 (0.65)	3.76 / 6.33	<0.001
	Model statistics	LR χ^2(7, n=2,025)=222.7, p<0.001, McFadden R^2=0.05		
Pain/Discomfort	CPC 2	2.00 (0.27)	1.54 / 2.60	<0.001
	Model statistics	LR χ^2(7, n=2,025)=107.85, p<0.001, McFadden R^2=0.02		

For peer review only - http://bmjopen.bmj.com/site/about/guidelines.xhtml

For peer review only

Anxiety/Depression	CPC 2	3.02 (0.42)	2.31 / 3.96	<0.001
	Model statistics	LR $\chi^2(7, n=2{,}024)=144.69$, $p<0.001$, McFadden $R^2=0.03$		

The adjusting co-variates are excluded from the table.

CPC=Cerebral Performance Category, EQ-5D-5L=EQ-5D descriptive system five-levels, IHCA=In Hospital Cardiac Arrest, OHCA=Out of Hospital Cardiac Arrest

For peer review only - http://bmjopen.bmj.com/site/about/guidelines.xhtml

Figure 1. Prevalence scoring of the EQ-5D-5L health dimensions, dichotomized into no problems (1) and problems (2-5), in relation to neurological outcome (good cerebral performance = CPC 1 and moderated cerebral disability = CPC 2).

For peer review only

For peer review only - http://bmjopen.bmj.com/site/about/guidelines.xhtml

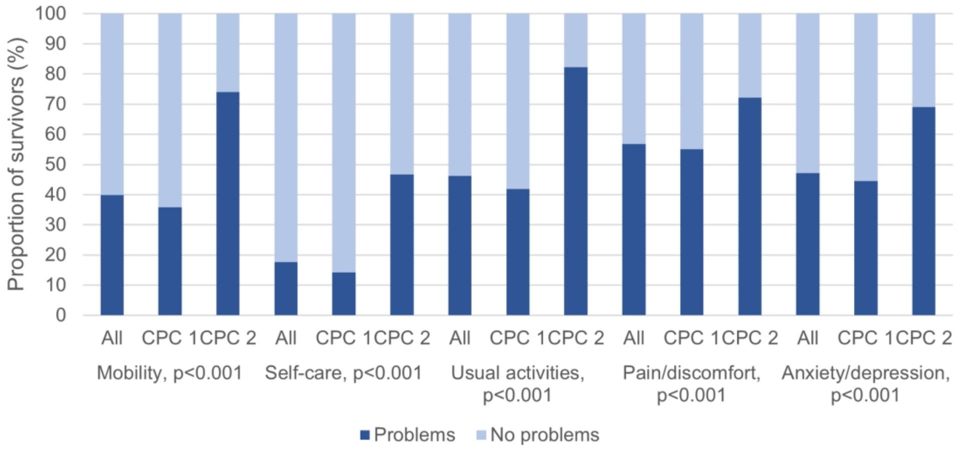

Figure 1. Prevalence scoring of the EQ-5D-5L health dimensions, dichotomized into no problems (1) and problems (2-5), in relation to neurological outcome (good cerebral performance = CPC 1 and moderated cerebral disability = CPC 2).

164x77mm (300 x 300 DPI)

STROBE Statement—Checklist of items that should be included in reports of ***cross-sectional studies***

	Item No	Recommendation	Page No
**Title and abstract**	1	(*a*) Indicate the study's design with a commonly used term in the title or the abstract	1
		(*b*) Provide in the abstract an informative and balanced summary of what was done and what was found	2
**Introduction**			
Background/rationale	2	Explain the scientific background and rationale for the investigation being reported	4-5
Objectives	3	State specific objectives, including any prespecified hypotheses	5
**Methods**			
Study design	4	Present key elements of study design early in the paper	5
Setting	5	Describe the setting, locations, and relevant dates, including periods of recruitment, exposure, follow-up, and data collection	5-6
Participants	6	(*a*) Give the eligibility criteria, and the sources and methods of selection of participants	6
Variables	7	Clearly define all outcomes, exposures, predictors, potential confounders, and effect modifiers. Give diagnostic criteria, if applicable	6-7
Data sources/ measurement	8*	For each variable of interest, give sources of data and details of methods of assessment (measurement). Describe comparability of assessment methods if there is more than one group	*N/A*
Bias	9	Describe any efforts to address potential sources of bias	8
Study size	10	Explain how the study size was arrived at	N/A
Quantitative variables	11	Explain how quantitative variables were handled in the analyses. If applicable, describe which groupings were chosen and why	7-8
Statistical methods	12	(*a*) Describe all statistical methods, including those used to control for confounding	7-8
		(*b*) Describe any methods used to examine subgroups and interactions	7-8
		(*c*) Explain how missing data were addressed	N/A
		(*d*) If applicable, describe analytical methods taking account of sampling strategy	N/A
		(*e*) Describe any sensitivity analyses	N/A
**Results**			
Participants	13*	(a) Report numbers of individuals at each stage of study—eg numbers potentially eligible, examined for eligibility, confirmed eligible, included in the study, completing follow-up, and analysed	8
		(b) Give reasons for non-participation at each stage	6
		(c) Consider use of a flow diagram	N/A
Descriptive data	14*	(a) Give characteristics of study participants (eg demographic, clinical, social) and information on exposures and potential confounders	Table 1.
		(b) Indicate number of participants with missing data for each variable of interest	Table 1-5
Outcome data	15*	Report numbers of outcome events or summary measures	9-10

For peer review only - http://bmjopen.bmj.com/site/about/guidelines.xhtml

Main results	16	(*a*) Give unadjusted estimates and, if applicable, confounder-adjusted estimates and their precision (eg, 95% confidence interval). Make clear which confounders were adjusted for and why they were included.	Table 3,5 and p 8,9,10
		(*b*) Report category boundaries when continuous variables were categorized	Figure 1, p 7
		(*c*) If relevant, consider translating estimates of relative risk into absolute risk for a meaningful time period	N/A
Other analyses	17	Report other analyses done—eg analyses of subgroups and interactions, and sensitivity analyses	6
**Discussion**			
Key results	18	Summarise key results with reference to study objectives	11-13
Limitations	19	Discuss limitations of the study, taking into account sources of potential bias or imprecision. Discuss both direction and magnitude of any potential bias	13-14
Interpretation	20	Give a cautious overall interpretation of results considering objectives, limitations, multiplicity of analyses, results from similar studies, and other relevant evidence	14
Generalisability	21	Discuss the generalisability (external validity) of the study results	13
**Other information**			
Funding	22	Give the source of funding and the role of the funders for the present study and, if applicable, for the original study on which the present article is based	15

*Give information separately for exposed and unexposed groups.

Note: An Explanation and Elaboration article discusses each checklist item and gives methodological background and published examples of transparent reporting. The STROBE checklist is best used in conjunction with this article (freely available on the Web sites of PLoS Medicine at http://www.plosmedicine.org/, Annals of Internal Medicine at http://www.annals.org/, and Epidemiology at http://www.epidem.com/). Information on the STROBE Initiative is available at www.strobe-statement.org.

For peer review only
